# Sarcomatoid Lung Carcinoma Successfully Treated With Combined Cytotoxic Chemotherapy and Anti-Programmed Death-Ligand 1 (Anti-PD-L1) Antibodies

**DOI:** 10.7759/cureus.95482

**Published:** 2025-10-27

**Authors:** Kohei Yoshimine, Kazunori Tobino, Hiroaki Ota, Yuri Hiramatsu, Ryuta Yamamoto

**Affiliations:** 1 Respiratory Medicine, Iizuka Hospital, Iizuka, JPN

**Keywords:** atezolizumab, carboplatin and paclitaxel, combination of chemotherapy and anti-pd-l1 antibody, lung cancer, sarcomatoid lung carcinoma

## Abstract

Pulmonary sarcomatoid carcinoma is a rare, aggressive subtype of non-small-cell lung cancer with a poor prognosis and no established standard therapy for advanced disease. We report the case of a 68-year-old man with stage IVB pleomorphic carcinoma whose tumor expressed programmed death-ligand 1 (PD-L1) with a tumor proportion score of 25%. The patient received four cycles of first-line therapy combining atezolizumab with carboplatin and paclitaxel. This treatment resulted in a significant partial response, with marked shrinkage of the primary tumor and metastatic lesions. This case suggests that the combination of an anti-PD-L1 antibody and platinum-based chemotherapy is a promising and effective therapeutic strategy for this challenging malignancy.

## Introduction

Lung cancer is a leading cause of cancer-related mortality worldwide. Pulmonary sarcomatoid carcinoma (PSC) is a rare group of non-small cell lung carcinomas (NSCLCs), accounting for approximately 0.3% to 1.3% of all pulmonary malignancies [1]. According to the World Health Organization classification, this entity is defined as a poorly differentiated NSCLC containing at least 10% sarcomatoid components, which are characterized by spindle and/or giant cells [1].

Consistent with their aggressive biology, these tumors are associated with a poor prognosis, and a standard of care for advanced or metastatic disease has not been established [1]. Historically, outcomes with conventional platinum-based chemotherapy have been unsatisfactory [2]. More recently, therapy with immune checkpoint inhibitors (ICIs) has emerged as a promising strategy, supported by findings that a high percentage of sarcomatoid carcinomas show significant PD-L1 expression [3]. However, while pivotal trials have established chemo-immunotherapy as a standard for common types of NSCLC, specific data on the efficacy of this combination in the rare sarcomatoid carcinoma subtype are still limited.

Here, we report a case of advanced sarcomatoid carcinoma of the lung (pleomorphic carcinoma, cT4N3M1c, stage IVB). The patient was treated with a combination of cytotoxic chemotherapy, consisting of carboplatin and paclitaxel, and the anti-PD-L1 antibody atezolizumab. This first-line therapy resulted in a significant clinical response, suggesting that this combination may be a viable treatment option for this rare and aggressive malignancy.

## Case presentation

A 68-year-old Japanese man was referred to our hospital for a detailed examination of a chest X-ray abnormality. He was asymptomatic, and his Eastern Cooperative Oncology Group performance status was 0. His medical history included type 2 diabetes, gallstones, and a history of surgical repair for an inguinal hernia and a perforated gastric ulcer. He had a 36-pack-year smoking history. Laboratory tests showed an elevated serum carcinoembryonic antigen level. Additionally, serum alkaline phosphatase (ALP) and lactate dehydrogenase (LDH) were elevated, which were considered to be a reflection of the tumor and bone metastasis. Other parameters were within their normal ranges (Table 1).

**Table 1:** Results of laboratory tests

Test	Res U/Lt	Reference range
White blood cells	5550 /μL	3300-8600 /μL
Neut	39.7%	39.5-74.5 %
Lym	37.4%	20.9-54.1 %
Mono	6.7%	3.6-9.8 %
Eos	2.4%	0.0-8.1 %
Red blood cells	3.99×10^6 /μL	4.35-5.55×10^6 /μL
Hemoglobin	14.1 g/dL	13.7-16.8 g/dL
Hematocrit	41.8%	40.7-50.1 %
Mean corpuscular volume	104.7 fL	83.6-98.2 fL
Platelet	22.1×10^3 /μL	15.8-34.8×10^3 /μL
Aspartate amonitransferase	19 U/L	13-30 U/L
Alanine aminotransferase	19 U/L	10-42 U/L
Lactate dehydrogenase	230 U/L	124-222 U/L
Alkaline phosphatase	238 U/L	38-113 U/L
γ-Glutamyl transpeptidase	40 U/L	13-64 U/L
Total bile acid	0.8 mg/dL	0.4-1.5 mg/dL
Albumin	4.3 g/dL	4.1-5.1 g/dL
Urea nitrogen	16 mg/dL	8-20 mg/dL
Creatinine	0.85 mg/dL	0.65-1.07 mg/dL
Sodium	142 mEq/L	138-145 mEq/L
Potassium	5.1 mEq/L	3.6-4.8 mEq/L
Chloride	104 mEq/L	101-108 mEq/L
Calcium	9.2 mg/dL	8.8-10.1 mg/dL
Glucose	105 mg/dL	73-109 mg/dL
C-reactive protein	0.11 mg/dL	0-0.14 mg/dL
HbA1c	7.00%	4.9-6.0 %
Prothrombin time %	137%	80-120 %
Prothrombin time-international normalized ratio	1.42 %	0.90-1.20 %
Activated partial thromboplastin time	24.5 sec	24-32 sec
Carcinoembryonic antigen	6.0 ng/ml	0-5 mg/ml
Cytokeratin 19 fragment	2.2 ng/ml	0-3.5 mg/ml
Pro-gastrin-releasing peptide	59.8 mg/mL	0-81 mg/ml
Hepatitis B virus Hep B surface antigen, Hep B surface antibody	Non-reactive, Non-reactive	Non-reactive, Non-reactive

**Figure 1:**
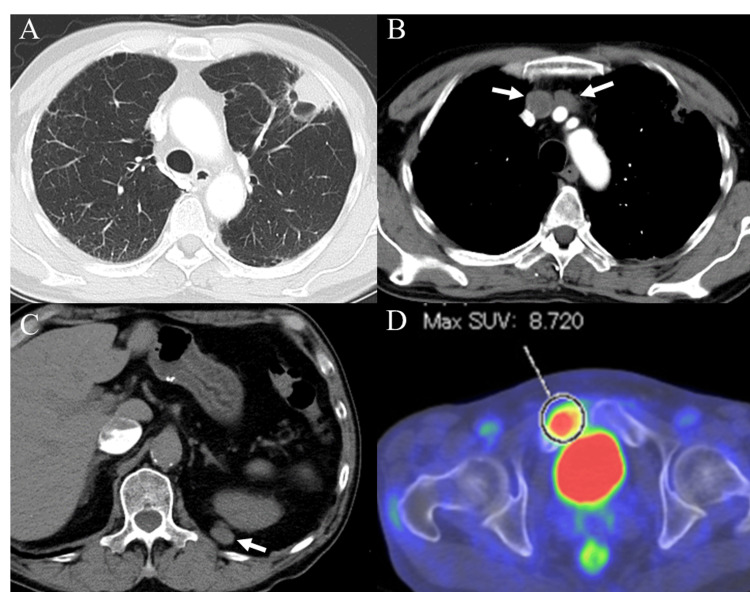
Initial imaging findings Contrast-enhanced whole body CT scan revealed a nodule in contact with the chest wall in the left upper lung lobe (A), multiple mediastinal lymphadenopathies (B, white arrows), and retroperitoneal metastases (C, white arrow). FDG PET/CT showed intense uptake of FDG in the pubic bone metastasis (D). FDG: F-18 fluorodeoxyglucose; PET/CT: positron emission tomography/computed tomography

Contrast-enhanced whole-body computed tomography (CT) revealed a 27×25-mm nodule in contact with the chest wall in the left upper lung lobe, multiple mediastinal lymphadenopathies, and retroperitoneal metastases (Figures 1A-1C). Additionally, F-18 fluorodeoxyglucose positron emission tomography/computed tomography (F-18 FDG PET/CT) showed increased FDG uptake in a pubic bone metastasis. The maximum standardized uptake value of FDG was 8.72 (Figure 1D).

**Figure 2:**
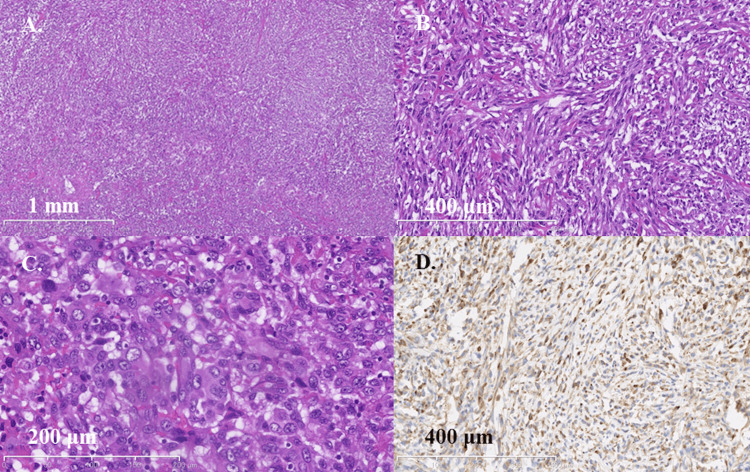
Pathological findings of the lung tumor specimen (A) Hematoxylin-Eosin (H&E) staining shows a dense proliferation of tumor cells (low magnification). (B) The tumor is composed of atypical, spindle-shaped cells (H&E staining, high magnification). (C) Multinucleated giant cell-like cells are also observed within the tumor (H&E staining, high magnification). (D) Immunohistochemical staining reveals that the tumor cells are positive for the cytokeratin marker AE1/AE3, confirming their epithelial origin.

An ultrasonography-guided percutaneous lung nodule biopsy was inconclusive. Therefore, a subsequent biopsy was performed via video-assisted thoracoscopic surgery. Pathological examination of the specimen from the left upper lung nodule revealed partially spindle-shaped tumor cells (Figures 2A-2B) and included multinucleated giant cell-like cells (Figure 2C). On immunohistochemistry, these cells were positive for AE1/AE3 (Figure 2D) and CAM5.2, but negative for p40.

Based on these findings, the patient was diagnosed with sarcomatoid carcinoma of the lung, specifically pleomorphic carcinoma (cT4N3M1c, stage IVB). Genetic testing for driver mutations, including EGFR mutations, EML-ALK fusions, and ROS1 mutations, was negative. The PD-L1 expression in the tumor cells (tumor proportion score: TPS) was 25%.

**Figure 3:**
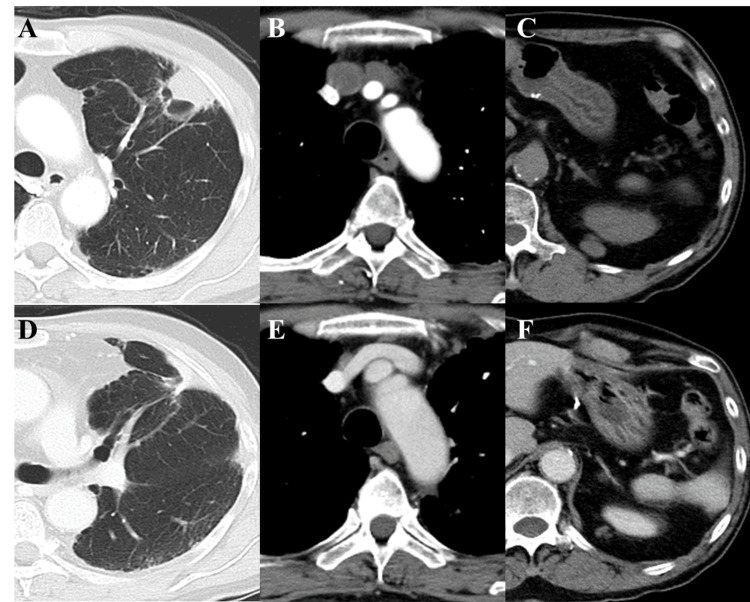
Treatment response Comparison of CT images before (A-C) and after (D-F) treatment. Lesions have improved significantly.

The patient received four cycles of atezolizumab (1200 mg) with carboplatin (area under the curve (AUC)=6) and paclitaxel (100 mg/m2) as first-line therapy. After completing four cycles of combination therapy over approximately three months, CT imaging confirmed a partial response, with marked shrinkage of both the primary tumor and metastatic lesions (Figure 3). The patient subsequently received six cycles of maintenance therapy with atezolizumab, administered every three weeks, over the next four and a half months. However, follow-up imaging revealed disease progression. The progression-free survival (PFS) was 8.1 months. During the treatment course, the patient experienced several adverse events, including Grade 4 neutropenia, Grade 3 thrombocytopenia, Grade 2 peripheral neuropathy, alopecia, and diarrhea. These toxicities primarily occurred during the induction phase. Supportive measures, such as granulocyte colony-stimulating factor (G-CSF) administration, transfusions, and dose reductions, were implemented as needed, allowing the continuation of therapy without discontinuation.

## Discussion

We report a case of advanced PSC that showed a significant response to first-line therapy combining the anti-PD-L1 antibody atezolizumab with carboplatin and paclitaxel. Notably, this favorable outcome was achieved in a patient whose tumor had a moderate PD-L1 tumor proportion score of 25%. Given the rarity of this disease and the lack of an established standard of care, these findings provide valuable clinical evidence supporting this chemo-immunotherapy regimen as a viable treatment strategy.

The significant clinical response observed in our patient treated with atezolizumab, carboplatin, and paclitaxel aligns with emerging evidence that establishes chemo-immunotherapy as a highly effective approach for advanced PSC. Historically, this disease has been challenging to treat with limited success from conventional chemotherapy. However, recent large-scale, real-world data confirm that adding an ICI to chemotherapy is associated with significantly longer progression-free and overall survival compared to chemotherapy alone [4]. Furthermore, our result is supported by prospective data from a Phase II trial, which demonstrated that first-line ICI combined with chemotherapy has robust efficacy and an acceptable safety profile in this specific patient population [5]. Therefore, our case contributes to the growing evidence that this combination regimen represents a primary therapeutic strategy for patients with advanced sarcomatoid carcinoma.

A particularly noteworthy aspect of this case is that a significant clinical response was achieved even though the tumor's PD-L1 expression was moderate, with a TPS of 25%. This is significant because improved outcomes with ICIs in PSC are often associated with high PD-L1 expression, such as a TPS of 50% or greater [3]. However, the strong response in our patient suggests that PD-L1 expression level alone may not be the sole determinant of ICI efficacy in this disease. It has been reported that these tumors are often characterized by an "immune-inflamed" phenotype, with features such as CD8+ T-cell infiltration, which provides a biological basis for a high sensitivity to immunotherapy [6]. While immunohistochemical analysis of CD8+ T-cell infiltration would have provided further support for this phenotype, such staining was not feasible in this case due to the retrospective nature of the report and the limited availability of tumor tissue. We acknowledge this as a limitation of the study. Therefore, our findings suggest that the inherent immunogenic nature of sarcomatoid carcinoma might allow for favorable responses to chemo-immunotherapy even in the absence of high PD-L1 expression, highlighting that patients in this subgroup should not be excluded from this therapeutic option.

The successful outcome in our case further solidifies the role of first-line chemo-immunotherapy in advanced PSC, but it also places this strategy within a broader, evolving treatment landscape. The utility of combining immunotherapy with chemotherapy is not confined to metastatic disease; similar success has been reported in the neoadjuvant setting for locally advanced PSC, where this approach has led to high rates of pathological response [7]. However, this does not imply a one-size-fits-all strategy. The dramatic response of a BRAF V600E-mutated PSC to targeted inhibitors highlights the critical need for comprehensive genomic profiling to identify patients who may benefit from alternative, personalized treatments [8]. Furthermore, for patients who may not be ideal candidates for standard PD-L1 pathway inhibition, such as those with PD-L1-negative tumors, more intensive strategies combining dual checkpoint blockade (anti-CTLA-4 and anti-PD-1) with chemotherapy have shown promise for achieving long-term responses [9]. Thus, a key clinical implication is the growing need to stratify PSC patients by stage, genomic alterations, and immune biomarkers to select the most appropriate therapeutic strategy.

## Conclusions

We have presented a case of advanced PSC that demonstrated a significant clinical response to first-line treatment with atezolizumab, carboplatin, and paclitaxel. This case reinforces the growing consensus that chemo-immunotherapy is an effective strategy for this aggressive disease, and it further suggests that a favorable response is possible even in tumors without high PD-L1 expression, likely due to the inherently immunogenic nature of PSC. While this outcome is encouraging, we must strongly emphasize the inherent limitations of a single case report. The favorable response observed in this one patient cannot be generalized to the broader population of patients with PSC, and these findings must therefore be interpreted with caution. The continued success in treating this rare malignancy will ultimately depend on a deeper understanding of its biological heterogeneity. Therefore, large-scale prospective studies are essential not only to confirm the efficacy and safety of this combination therapy but also to identify robust predictive biomarkers that can guide a more personalized treatment approach for every patient.
